# Ly49 Receptors: Innate and Adaptive Immune Paradigms

**DOI:** 10.3389/fimmu.2014.00145

**Published:** 2014-04-02

**Authors:** Mir Munir A. Rahim, Megan M. Tu, Ahmad Bakur Mahmoud, Andrew Wight, Elias Abou-Samra, Patricia D. A. Lima, Andrew P. Makrigiannis

**Affiliations:** ^1^Department of Biochemistry, Microbiology and Immunology, University of Ottawa, Ottawa, ON, Canada; ^2^College of Applied Medical Sciences, Taibah University, Madinah Munawwarah, Kingdom of Saudi Arabia; ^3^Biomedical and Molecular Sciences, Queen’s University, Kingston, ON, Canada

**Keywords:** Ly49, innate immune receptors, NK cells, innate immunity, receptor function

## Abstract

The Ly49 receptors are type II C-type lectin-like membrane glycoproteins encoded by a family of highly polymorphic and polygenic genes within the mouse natural killer (NK) gene complex. This gene family is designated *Klra*, and includes genes that encode both inhibitory and activating Ly49 receptors in mice. Ly49 receptors recognize class I major histocompatibility complex-I (MHC-I) and MHC-I-like proteins on normal as well as altered cells. Their functional homologs in humans are the killer cell immunoglobulin-like receptors, which recognize HLA class I molecules as ligands. Classically, Ly49 receptors are described as being expressed on both the developing and mature NK cells. The inhibitory Ly49 receptors are involved in NK cell education, a process in which NK cells acquire function and tolerance toward cells that express “self-MHC-I.” On the other hand, the activating Ly49 receptors recognize altered cells expressing activating ligands. New evidence shows a broader Ly49 expression pattern on both innate and adaptive immune cells. Ly49 receptors have been described on multiple NK cell subsets, such as uterine NK and memory NK cells, as well as NKT cells, dendritic cells, plasmacytoid dendritic cells, macrophages, neutrophils, and cells of the adaptive immune system, such as activated T cells and regulatory CD8^+^ T cells. In this review, we discuss the expression pattern and proposed functions of Ly49 receptors on various immune cells and their contribution to immunity.

## Introduction

**Figure 1:**
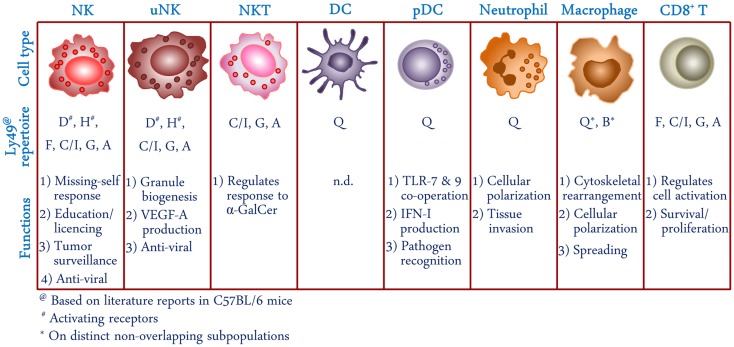
**Schematic representation of cell types expressing Ly49 receptors**. Receptor repertoire and proposed functions of Ly49 in different cellular subsets is shown based on literature reports in C57BL/6 mice. NK, natural killer; uNK, uterine natural killer; NKT, natural killer T; DC, dendritic cells; pDC, plasmacytoid dendritic cell; α-GalCer, α-galactosylceramide; VEGF-A, vascular endothelial growth factor A; TLR, Toll-like receptor; IFN-I, type I interferon; n.d., not determined.

Major histocompatibility complex (MHC) recognition is central to both innate and adaptive immune recognition. The function of both the innate immune effectors, such as natural killer (NK) cells, and the adaptive immune effectors, such as T cells, depend upon the recognition of MHC-I molecules expressed on aberrant cells; however, the mode of recognition varies between the two lymphocyte subsets. T cell receptors (TCR) have specificity to the antigenic peptide bound to MHC-I and form contacts to the peptide as well as to the MHC molecule (1, 2). NK cell receptors, such as the Ly49 in mice, bind MHC-I molecules in a peptide-dependent but not peptide-specific manner (3). An exception is the Ly49C, a member of the Ly49 receptor family (Table 1), whose binding to H-2K^b^ haplotype appears to be peptide-specific (3, 4). The binding interface between Ly49 and MHC-I, revealed from crystal structures of Ly49A and H-2D^b^ molecules, is distinct and away from the peptide binding groove on MHC-I (5). While signals downstream of TCR engagement specifically activate T cell functions, the Ly49 receptors can be activating or inhibitory in nature, and their expression is not limited to NK cells. In addition to NK cells, other leukocytes have also been shown to express inhibitory Ly49 receptors, such as the CD8^+^ T cells, CD3^+^ cells, intestinal epithelial lymphocytes (IELs), NKT cells, uterine NK cells (uNK) cells, and cells of the myeloid lineage (6–12). Here, we review the expression of Ly49 receptors on different cells of the innate and adaptive immune system (Figure 1), and their contribution to immunity.

**Table 1:** **Mouse Ly49 and human KIR receptors for MHC-I**.

Mouse^a^				Human	Function
NOD	129	B6	BALB	
Ly49D	Ly49P	Ly49D	Ly49L	KIR2DL4	Activating
Ly49H	Ly49R	Ly49H		KIR2DS1	
Ly49M	Ly49U			KIR2DS2	
Ly49P_1_				KIR2DS3	
Ly49P_3_				KIR2DS4	
Ly49U				KIR2DS5	
Ly49W				KIR3DS1	
Ly49A	Ly49B^b^	Ly49A	Ly49A	KIR2DL1	Inhibitory
Ly49B^b^	Ly49E	Ly49B^b^	Ly49B^b^	KIR2DL2	
Ly49C	Ly49EC_2_	Ly49C	Ly49C	KIR2DL3	
Ly49E	Ly49G	Ly49E	Ly49E	KIR2DL5	
Ly49F	Ly49I_1_	Ly49F	Ly49G	KIR3DL1	
Ly49G_2_	Ly49O	Ly49G	Ly49I	KIR3DL2	
Ly49I	Ly49Q_1_	Ly49I	Ly49Q		
Ly49Q	Ly49S	Ly49J			
	Ly49T	Ly49Q			
	Ly49V				

*^a^*Ly49 pseudogenes* do not encode any functional product and are not included*.

*^b^ Encoded by a gene outside the *Ly49* gene cluster*.

## Ly49 Receptors

**Figure 2:**
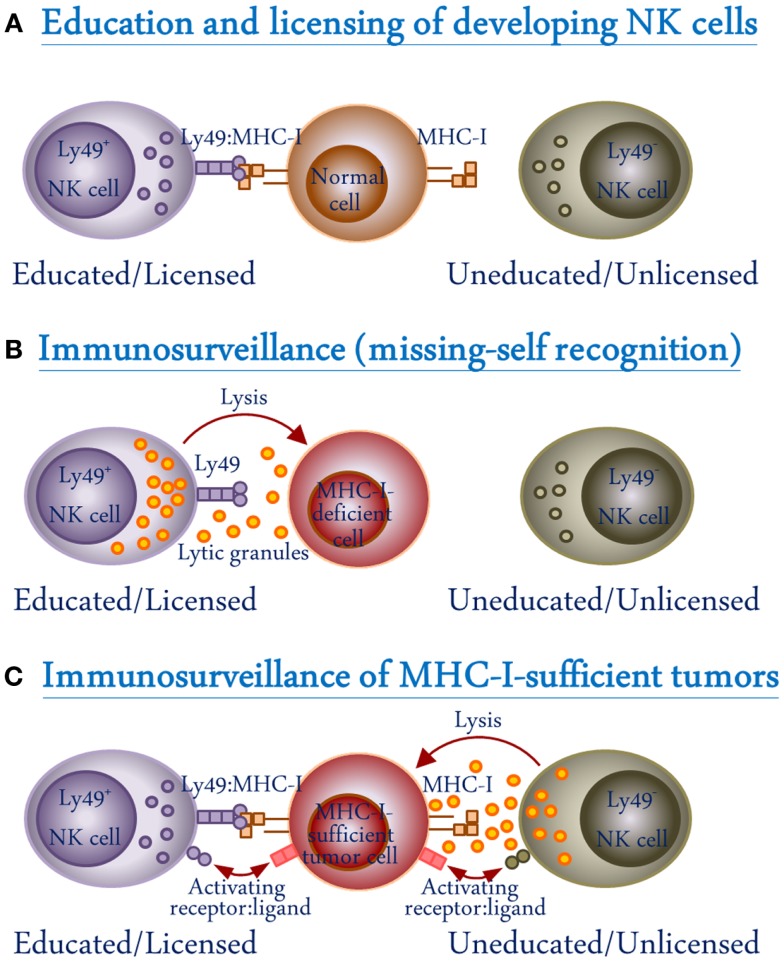
**Schematic representation of the role of Ly49 receptors in NK cell development and function**. **(A)** During NK cell development, interactions between the inhibitory Ly49 receptors and their self-MHC-I ligands on normal cells result in NK cell functional maturation (education/licensing). **(B)** Licensed Ly49^+^ but not unlicensed Ly49^−^ NK cells recognize MHC-I-deficient cells and kill them through the release of lytic granules (missing-self recognition). **(C)** Tumor cells express ligands which are recognized by activating receptors on NK cells. However, MHC-I-expressing tumor cells can inhibit licensed NK cells through interactions with their inhibitory Ly49 receptors. Unlicensed NK cells will not be inhibited in this way because they lack Ly49 receptors.

The innate MHC-I receptors include the human killer cell immunoglobulin-like receptor (KIR), NKG2/CD94, and mouse Ly49 families of receptors. The genes that encode the mouse MHC-I receptors are clustered together in the natural killer gene complex (NKC) on mouse chromosome 6. The Ly49 receptors are homodimeric type II glycoproteins of the C-type lectin-like superfamily. They are structurally distinct but functionally similar to human KIR in terms of MHC-I recognition, and therefore provide a useful model system to study the role of this class of receptors in immune regulation. A list of activating and inhibitory Ly49 receptors in different mouse strains and KIR in humans is shown in Table 1. MHC-I receptors generally inhibit NK cell function when they are engaged by self-MHC ligands. Therefore, inhibitory Ly49 receptors are generally agreed to be important for the prevention of autoimmunity by suppressing NK cell activation. The acquisition of inhibitory Ly49 for self-MHC-I is also a key step in the “licensing” of developing NK cells to avoid a hyporesponsive state (Figure 2A) (13). In contrast, the activating Ly49 receptors recognize ligands that are expressed on abnormal or infected cells, and activate cytokine production and cellular cytotoxicity by NK cells. The integration of signals from the activating and inhibitory Ly49 receptors ultimately determines the functionality of NK cells.

### Intracellular signaling

The inhibitory and activating members of Ly49 and KIR families are characterized by the presence or absence of immunoreceptor tyrosine-based inhibitory motif (ITIM) domains in their cytoplasmic tail (14–16). NK cell stimulation results in differential phosphorylation of Ly49 receptors. Mason and co-workers first reported that tyrosine phosphorylation was restricted to the inhibitory Ly49 molecules such as Ly49 A, C/I, and G2, while the activating Ly49D was not phosphorylated (17). The phosphorylated Ly49 molecules were shown to be associated with the src homology 2 (SH2) domain-containing protein phosphatase SHP-1 in this study (17). Phosphorylation of the tyrosine residue within the ITIM is responsible for the recruitment and activation of SH2 domain-containing protein tyrosine phosphatases (18). Although SHP-1 appears to be the major phosphatase required for Ly49-mediated inhibition, it may not be the only inhibitory mediator since Ly49A function is diminished but not completely absent in mice deficient in SHP-1 (19). Although engagement of inhibitory receptors results in ITIM phosphorylation, it is greatly enhanced when it is cross-linked to an activating receptor complex (20, 21). This demonstrates an elegant regulatory mechanism whereby inhibitory receptor phosphorylation induced by engagement of activating receptors results in recruitment of protein tyrosine phosphatases, and the subsequent suppression of tyrosine phosphorylation-based signals downstream of the activating receptors. In contrast to the inhibitory receptors, the activating Ly49 receptors transduce signals through associated adaptor proteins, such as DAP12, which possess an immunoreceptor tyrosine-based activation motif (ITAM) (22–24). This interaction is mediated by a charged residue (arginine) in the transmembrane segment of Ly49, as was shown for Ly49D (25). Cross-linking of activating Ly49 leads to phosphorylation of tyrosine residues within the ITAM of DAP12 as well as phosphorylation of other proteins, including members of the Src-family of tyrosine kinases, the mediators of downstream signaling events (26).

### Genetic diversity

**Figure 3:**
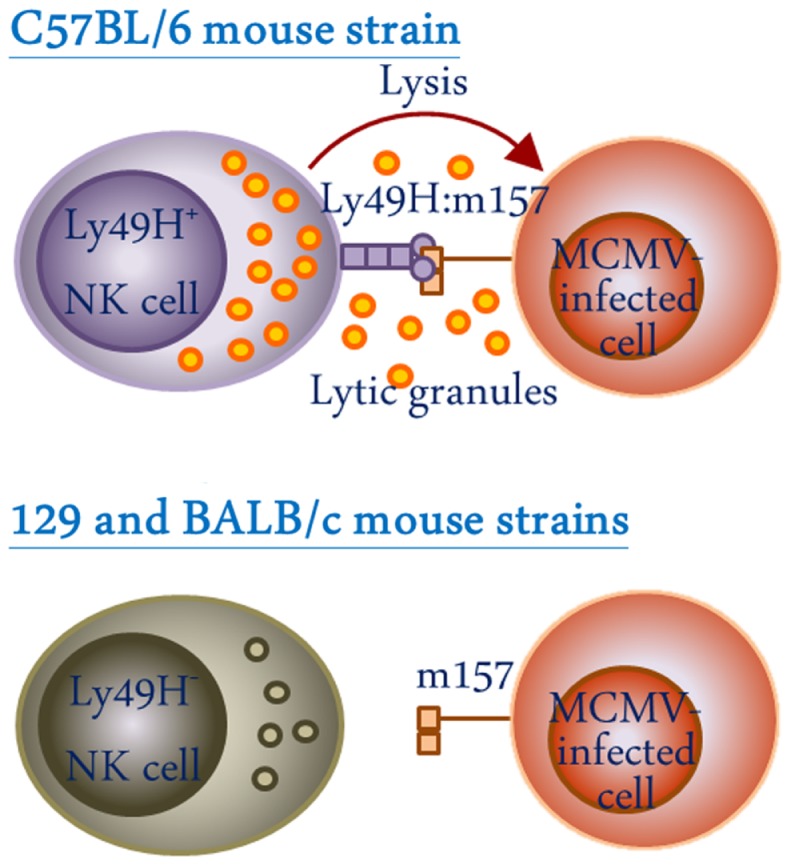
**Schematic representation of the role of the activating Ly49H receptors in recognition of MCMV-infected cells**. NK cells from C57BL/6 but not 129 or BALB/c mouse strains express Ly49H and are capable of recognizing m157 viral protein on the surface of MCMV-infected cells, thereby, conferring resistance against MCMV infection.

One of the striking features of Ly49 receptors is the polymorphic and polygenic nature of the *Ly49* gene cluster among the inbred mouse strains (27–30). This results in heterogeneity in both the type and level of Ly49 molecules expressed in different mouse strains (Table 1) (31). Among the four inbred mouse strains whose *Ly49* gene haplotypes have been extensively characterized, the BALB/c mouse strain possess the smallest haplotype with 8 genes (32), and the NOD/ShiLtJ strain possess the largest haplotype with 21 discernable genes (33). The C57BL/6 haplotype, which was the first to be characterized, and the 129 mouse strain haplotype possess 15 and 19 genes, respectively (28, 29, 34–36). In all the characterized *Ly49* haplotypes, there appears to be a limited degree of conservation in the form of “framework” genes which delineate the regions of variable numbers of strain-specific genes. The framework gene pairs in mice are *Ly49q-e, Ly49i-g*, and *Ly49c-a*. *Ly49* haplotype diversity has evolved as a result of multiple duplication and deletion events. Examples of strain-specific Ly49 gene inactivation are evident in different strain of mice mostly mediated by stop codons within the coding region (37–39). Such diversity is possibly driven by selective pressure due to pathogenic challenge, since NK cells appear to be critical for the control of viral infections (40, 41). In mice, the activating Ly49H receptor directly interacts with the murine cytomegalovirus (MCMV) *m157* gene product on NK cells and confers resistance to MCMV in the C57BL/6 mouse strain which possesses the *Ly49h* gene (Figure 3). Conversely, 129 and BALB/c strains lack the gene for activating Ly49H and hence are highly susceptible to MCMV infection (42–47).

## Ly49 Expression and Function on NK Cells

Ly49 receptors are best known for their role in the regulation of NK cell functions. Both the activating and inhibitory Ly49 receptors are expressed by NK cells (Figure 1). Murine NK cells express up to six members of the Ly49 receptor family in an overlapping fashion, such that the average NK cell expresses two to three Ly49 receptors (48). Ly49 expression begins early during NK cell development in the bone marrow (BM). An *in vivo* differentiation study demonstrated that there are five stages of NK cell development in murine BM, as assessed by surface expression of NK cell markers (49). In the first three stages of development, NK cells sequentially attain expression of CD122 (stage I; CD122^+^ NK1.1^−^ DX5^−^ Ly49^−^), then NK1.1, DX5, and NKG2A (stage II; CD122^+^ NK1.1^+^ DX5^lo^ CD94/NKG2A^+^ Ly49^−^), and finally Ly49 at stage III. Afterward, immature NK cells undergo extensive cell division and expansion in stage IV followed by functional maturation at stage V of their development (49). Ly49E is the only member of Ly49 family that is expressed on fetal NK cells and its expression is lost early after birth (50). Expression of other Ly49 family members are detected on developing NK cells during the first 2–3 weeks after birth and reach optimal levels within 6–8 weeks after birth (50, 51).

### NK cell education and target cell recognition

Ly49 receptors play an important role in NK cell education and recognition of target cells. The mechanism behind NK cell recognition of target cells was discovered by Kärre and colleagues, and accordingly named the “missing-self hypothesis” (Figure 2B) (52). According to the hypothesis, NK cells survey MHC-I expression on cells with which they come into contact (52). Abnormal or infected cells often down-regulate expression of MHC-I on their surface in order to avoid detection and killing by cytotoxic T cells; however, this down-regulation is recognized by, and functions as a kill signal for NK cells (52). In this seminal work, the RBL-5 lymphoma cell line was mutagenized to derive two new cell lineages, an MHC-I-expressing RMA and MHC-I-deficient RMA-S. It was observed that the MHC-I-deficient RMA-S cells, following injection into mice, were rapidly eliminated while the MHC-I-expressing RMA cells were able to develop into tumors (52). This “missing-self” hypothesis was further supported by the finding that cells isolated from mice lacking expression of β_2_m, the light chain component of MHC-I that is necessary for its surface expression, were readily recognized and killed by NK cells, while re-introduction of a β_2_m transgene restored the resistance to NK cell killing (53). In addition to the increased susceptibility of cells isolated from MHC-I-deficient mice to NK cell killing, it was also noted that NK cells from β_2_m^−/−^ mice exhibited a diminished ability to kill traditional NK cell targets, when compared to wild-type (WT) mice (53). This led to the proposal that MHC-I interactions during NK cell development are needed for induction of NK cytolytic activity (Figure 2A). This hypothesis was later substantiated by the discovery that NK cells must undergo an MHC-I-dependent “licensing” process in order to be functional, in which a self-specific Ly49 receptor interacts with self-MHC-I (13). Similarly, we have demonstrated that the NK cells from Ly49-deficient mice are unlicensed and show impaired missing-self response against MHC-I-deficient target cells (54). This education requirement has also been found in human NK cells with the interaction of MHC-I and KIR (55, 56). The licensed NK cells are functionally active while at the same time self-tolerant due to recognition of MHC-I through their inhibitory Ly49 receptors. In contrast, the unlicensed NK cells – which lack self-MHC-specific inhibitory receptors – are tolerant due to their hyporesponsive nature.

The mechanics of the education process are still unknown, with various contested models. While education was originally thought to occur during NK cell development in the BM (13), adoptive transfer studies utilizing splenic NK cells from MHC-I deficient mice into WT hosts were able to restore NK functionality, thus showing that education does not occur only during development of immature NK cells, but is actually a dynamic and reversible process (57, 58). The arming and disarming model are the most widely accepted models thus far. Based on the arming model, NK cells which lack expression of an inhibitory self-MHC-I-specific Ly49 receptor are unable to fully mature into functional NK cells (become “armed”) and thus remain in their hyporesponsive state (59). In contrast, the disarming model proposes that NK cells which lack expression of an inhibitory self-MHC-I-specific Ly49 receptor, and thus are chronically stimulated due to lack of inhibitory signaling, become anergic and hyporesponsive to target cells (59).

The type of interaction between the Ly49 receptor and the MHC-I molecule during education has also been shown to be important and somewhat controversial: it is unknown whether the Ly49 is interacting in *cis* with MHC-I on the NK cell surface itself or in *trans* with the MHC-I expressed on another cell. *Trans-*mediated licensing is supported by various groups, using either adoptive transfer from MHC-I-deficient mice in WT recipients or doxycycline-inducible MHC-I expression in MHC-I-deficient β_2_m^−/−^ mice to observe interactions between MHC-I-deficient NK cells in an MHC-I-sufficient environment (57, 58, 60). On the other hand, using a modified Ly49A receptor, which is only capable of binding in *trans* with ligands on other cell surfaces but not in *cis*, Chalifour and colleagues found that NK cells from these mice did not appear to be fully educated (61). As well, Bessoles and colleagues’ recent work also suggests a *cis* interaction contribution to NK functionality, though the precise role is not clear (62).

### NK cell-mediated immunosurveillance

Major histocompatibility complex-I-dependent “missing-self” recognition by NK cells has great implications on the immunosurveillance of abnormal cells which have lost surface expression of MHC-I molecules. Lost or aberrant surface expression of MHC-I in human tumors is classified into seven phenotypes as follows: (1) total HLA loss, (2) HLA haplotype loss, (3) HLA locus loss, (4) HLA allelic loss, (5) compound phenotypes, (6) unresponsiveness to interferons (IFNs), and (7) gain of MHC-Ib (63). Altered MHC-I expression is highly prevalent in human tumors and various alterations have been associated with different cancer types: HLA haplotype loss is the most common alteration and has been described in laryngeal (64), non-Hodgkin’s lymphoma (65), and pancreatic cancer (66). Loss of MHC-I expression along with up-regulation of ligands for activating NK cell receptors on tumor cells results in their recognition and elimination by NK cells. We have shown that Ly49-deficient mice are unable to recognize and eliminate MHC-I-deficient target cells (54). On the other hand, the 5E6 antibody (to block the inhibitory Ly49I/C signaling) and 1-7F9 antibody (to block KIR signaling) have been shown to be useful in the treatment of leukemia and multiple myeloma in mice and humans, respectively; therefore, manipulation of human KIR signaling has been proposed as a potential cancer therapeutic (67–70). Finally, there is also evidence that a member of the Ly49 receptor family, Ly49A, recognizes the non-classical MHC-I molecule H2-M3 and in part mediates NK cell licensing (71). Ly49A^+^ NK cells from WT mice exhibit enhanced responsiveness compared to H2-M3-deficient mice. H2-M3-deficient mice display defective tumor control with increased B16F10 pulmonary metastatic burden, and increased incidence of MCA-induced fibrosarcoma (71, 72).

More recently, a paradigm shift was suggested following the study by Tarek and colleagues, wherein it was found that unlicensed NK cells are better at killing neuroblastoma tumors (73). Unlike the traditionally low levels of MHC-I expression on tumors, neuroblastoma cells express high levels of MHC-I, which can inhibit licensed NK cells. On the other hand, the unlicensed NK cells are not inhibited by MHC-I on neuroblastoma cells (Figure 2C). It was found that patients lacking HLA ligands for their KIRs showed improved prognosis and survival when treated with 3F8, an antibody targeting the disialoganglioside surface antigen, in contrast to the educated NK cells which were selectively inhibited by HLA ligands expressed on the tumor cells (73). In such case, the inhibitory signals through KIR-dependent recognition of HLA on the target cell dampen the beneficial effect of educated NK cells. Blocking this KIR interaction with MHC-I could prove therapeutically beneficial in such circumstances.

The Ly49 receptors on NK cells also play a role in immunity against virus infections. The importance of NK cells in innate defense against viral infections was highlighted by studies which have shown severe and recurrent infections by otherwise benign viruses in individuals lacking NK cell functions (40, 41). Viruses have developed several strategies to evade recognition of NK cells. This may occur by expression of viral proteins on infected cells that resemble host MHC-I molecules and inhibit NK cells through interaction with the inhibitory receptors, or by down-regulation of activating ligands on the surface of infected cells (74). The arms race between the viruses and NK cells has resulted in the selection of receptors for their ability to specifically recognize virus-infected cells. For instance, the activating Ly49H receptor on NK cells has evolved to recognize the glycoprotein m157, a MCMV-encoded structural homolog of MHC-I, on the surface of infected cells and imparts resistance in the C57BL/6 mouse strain (Figure 3) (46). Ly49H-deficient C57BL/6 mice and other mouse strains (129 and BALB/c), which lack the gene encoding for Ly49H, are highly susceptible to MCMV infection (43, 46, 47). The mutant MCMV strain that lacks m157 glycoprotein is significantly more virulent in C57BL/6 mice (75), indicating an important role for Ly49H in host defense against MCMV. Similarly, the activating Ly49P receptor protects MA/My mice from lethal MCMV infection through the recognition of MCMV-encoded m04 proteins in association with H-2D^k^ MHC-I haplotype (76). Mutant MCMV strains that lack m04 are lethal in MA/My mice since this mouse also lacks the activating Ly49H (77). Interestingly, Orr and colleagues have demonstrated that Ly49H^+^ NK cells that do not express inhibitory Ly49 receptors are more efficient in controlling MCMV infection than Ly49H^+^ NK cells that express inhibitory Ly49 receptors, possibly due to the lack of inhibitory signals in the former (78). Therefore, blocking inhibitory receptors on NK cells may have beneficial therapeutic effects in certain viral infections, as has been shown in cancers of humans and mice (67–70).

## Ly49 Expression and Function on Uterine NK Cells

Uterine NK cells (uNK) are resident lymphocytes of the uterus. In the non-pregnant mouse uterus, uNK cells are few and express CD122, CD49b (DX5), NKp46, CD11b, CD27, and CD69; a phenotype similar to peripheral blood and spleen NK cells (11, 79). These cells also express Ly49G2 (80, 81). During pregnancy, a massive influx of NK cells expressing Ly49 receptors occurs in the mouse decidualized uterus (11, 82–84). In the decidua, two subsets of uNK cells are identified based on the expression of *N*-acetyl-l-galactosamine, which can bind the *Dolichos biflorus agglutinin* (DBA) lectin: DBA^+^ and DBA^−^ subsets (11, 83). Both subsets are known to express Ly49 receptors (Figure 1) (11). DBA^−^ uNK cells originate from NK progenitors cells (Lin^−^CD122^+^NK1.1^−^) and like splenic NK cells, they are the source of decidual IFN-γ. However, their frequency remains constant throughout the pregnancy (11, 84, 85). DBA^+^ uNK cells are absent in the non-pregnant uterus, expand dramatically up to mid-gestation (gestational day 8.5–10.5), and decrease again in late pregnancy (11, 83, 86). The DBA^+^ uNK cells have a unique receptor repertoire which includes Ly49 receptors (11). Early in pregnancy, they perform potent vasculo-angiogenic activity demonstrating that they are active NK cells (85–90). The origin of DBA^+^ uNK cells is unclear, but uterus transplant experiments suggest that DBA^+^ uNK cells are recruited from the circulation (91).

The Ly49 receptor repertoires of DBA^+^ and DBA^−^ uNK cells differ. Ly49C/I and D are expressed at low levels and Ly49G2 is highly expressed on DBA^+^ compared to the DBA^−^ uNK cells, while Ly49A and H are expressed at similar levels (11). They seem to play an important role during mouse pregnancy. Antigenic disparity between parental H-2K genes (BALB/C: *d haplotype* and B6: *b haplotype*) affects the trophoblast-induced transformation of uterine vasculature (92). In the B6 mouse strain, the trophoblast giant cells express high levels of H-2K^b^ and H-2D^b^, the ligands for various Ly49 receptors (92–94). Our observation of pregnancy defects in the Ly49-knockdown mouse model and the works described above, suggest an important role of Ly49 receptors on uNK cells in mouse pregnancy.

In addition to their role in normal pregnancy, uNK cells can act as effector cells against infectious agents at the maternal–fetal interface. Human cytomegalovirus (HCMV) is the most common cause of intra-uterine and congenital virus infection (95). Human uNK cells have been shown to display cytotoxic effector function upon recognition of HCMV-infected cells (96). During mouse pregnancy, MCMV infection results in vascular dysfunction of mesenteric and uterine arteries (97). The activating Ly49H receptor is responsible for recognition and elimination of MCMV-infected cells (46). In addition, uNK cells express several vaso-active factors to regulate vaso-constriction and dilation (98), thereby making them potential modulators during normal and infectious pregnancy complications.

## Ly49 Expression and Function on NKT Cells

NKT cells are defined as CD1d-restricted T cells that express an invariant T cell antigen receptor, variable (V) and joining (J) Vα14Jα18 in mice, and Vα24Jα18 in humans, combined with a limited TCRβ chain repertoire (Vβ8.2, Vβ7, or Vβ2 in mice and Vβ11 in humans). These TCR αβ-chain combinations result in NKT cells with specificity for glycolipid antigens presented by the MHC-I-related CD1d surface protein (99, 100).

Murine TCRαβ^+^ NK1.1^+^ NKT cells are known to express other NK cell-associated molecules including CD122, CD16, DX5, CD94/NKG2, and the Ly49 family of receptors. Thus far, only inhibitory Ly49 receptors have been shown to be expressed on NKT cells (Figure 1) (101). One reason for this could be that, forced expression of Ly49D, an activating receptor, on T cells by transgenesis results in impaired thymocyte maturation (102). It has been shown that Ly49 expression inversely correlates with their response to α-galactosylceramide (α-GalCer), a potent stimulator of CD1d-restricted NKT cells. MHC-I-dependent α-GalCer presentation by dendritic cells (DC) stimulates Ly49^−^ but not Ly49^+^ NKT cells (12). On the other hand, MHC-I-deficient DCs effectively present α-GalCer to Ly49^+^ splenic NKT cells. When Ly49:MHC-I interactions are blocked using anti-Ly49 A, C/I, and G monoclonal antibodies (mAb), Ly49^+^ NKT cells were efficiently stimulated with α-GalCer-pulsed DCs, demonstrating inhibition of TCR-mediated activation signals by inhibitory Ly49:MHC-I interactions on NKT cells (101). Therefore, Ly49 receptors appear to have a similar role in regulating NKT cell responses as has been described for NK cells.

Expression of Ly49 and response to α-GalCer vary greatly among NKT cells from different anatomical sites. Almost 85% of splenic CD1d-independent NKT cells from β2m-deficient mice express Ly49A, C, G, or I, whereas only 50% of splenic NKT cells from WT mice express these Ly49 receptors (12). In the mouse thymus, over 75% of NKT cells stain with CD1d tetramers (103). Similarly, Oberg and colleagues have reported that almost 70% of thymic NKT cells express Ly49A, C, G, or I (12). Despite the high percentage of CD1d-restricted NKT cells in the thymus, thymic NKT cells are weakly stimulated with α-GalCer-pulsed DCs as compared with splenic NKT cells. The inability of most thymic NKT cells to respond to α-GalCer does not seem to be due to inhibition via Ly49:MHC-I interaction, because α-GalCer-pulsed MHC-I-deficient DCs also fail to stimulate thymic NKT cells (104). It seems likely that CD1d-restricted Ly49^+^ thymic NKT cells may be functionally immature and may constitute the precursors for mature CD1d-restricted NKT cells. Whereas thymic NKT cells are thought to be precursors of splenic CD1d-restricted NKT cells, the origin of NKT cells in BM is still unclear. Phenotypically, they are different from thymic NKT cells (105), and are thought to be extra-thymically derived (106). Almost 85% of BM NKT cells express Ly49A, C, G, or I, however, only 30% of them bind CD1d tetramers (103). Like their thymic counterparts, BM NKT cells do not respond to α-GalCer-pulsed MHC-I-sufficient and -deficient DCs. It remains to be determined whether all CD1d-restricted BM NKT cells are Ly49^+^, and whether they are immature NKT cells belonging to a separate NKT cell lineage.

## Ly49 Expression and Function on Cells of the Myeloid Lineage

While most members of Ly49 family of receptors are exclusively expressed on NK, NKT, and T cell subsets, Ly49Q and B have a unique pattern of expression and are found on distinct subsets of myeloid lineage cells (6, 8, 107). Ly49B is expressed on multiple distinct subpopulations of myeloid cells defined by CD11b and Gr-1 expression, with morphological characteristics of granulocytes and monocyte/macrophages (8). Ly49B expression was generally low in these cells and was up-regulated when cells were treated with lipopolysaccharide (LPS), IFN-α, and IFN-γ. Cells expressing Ly49B were reported to be more numerous in the Peyer’s patches and the lamina propria of the gut, implicating a role of Ly49B in gut immunobiology (8). Interestingly, Ly49B and Q were reported to be expressed on non-overlapping myeloid subpopulations among freshly isolated spleen and BM cells (8). Ly49Q is an inhibitory receptor and has been shown to associate with SHP-1/2 upon cross-linking (107). Its expression was detected on Gr-1^+^ cells in mouse fetal liver and adult BM and spleen. Expression was found to be higher on immature myeloid precursors and IFN-γ-treated macrophages (107). Ly49Q cross-linking on macrophage cell lines induces formation of cell polarity and spreading through cell cytoskeletal rearrangement (Figure 1) (107). Similarly, Ly49Q appears to be essential for cellular polarization and invasion of extravascular tissues by neutrophils during an inflammatory response (Figure 1) (108). In addition, Ly49Q expression is induced on osteoclasts which differentiate from the monocyte/macrophage lineage cells by receptor activator of nuclear factor-κB ligand (RANKL) treatment, and plays a role in osteoclastogenesis (109).

Another myeloid lineage cell type that expresses high levels of Ly49Q is the plasmacytoid dendritic cell (pDC) (Figure 1). Virtually all peripheral and the majority of BM pDCs express Ly49Q, which also correlates with the sequential development and activation of these cells (6, 7, 110). pDCs are specialized in direct virus recognition and secretion of large amounts of IFN-I early during infection (111, 112). Using a gene knock-out mouse model, we have shown that Ly49Q, despite being an inhibitory receptor, positively regulates Toll-like receptor (TLR)-mediated IFN-I production by pDCs (113, 114). In mice lacking Ly49Q, both TLR7 and TLR9-mediated IFN-I production by pDCs is attenuated (113). Although the precise mechanism of this positive regulation is not known, it is Ly49Q ITIM-dependent but may not involve interaction with the SHP-1/2 phosphatases (114). One of the possible mechanisms could be a cooperative activation of interferon regulatory factors (IRF) in conjunction with TLR-mediated downstream signals, leading to enhanced IFN-I production. Alternatively, Ly49Q may aid in the uptake of microbial macromolecules, such as unmethylated CpG oligodeoxynucleotides (ODN), and their trafficking to endosomal compartments where TLRs are located. We have demonstrated a specific down-regulation of Ly49Q from the pDC surface when treated with TLR9 agonist CpG ODN (114). This is similar to the down-regulation and co-internalization of human KIR3DL2 with CpG ODN in NK cells (115). A direct *in vitro* binding of CpG ODN to KIR3DL2 was also shown in this study. Co-localization of Ly49Q with CpG ODN in TLR9 containing endosome/lysosome compartments has previously been demonstrated, however it remains to be determined if Ly49Q is the sensor and distributor of CpG ODN to these compartments (116).

## Ly49 Expression and Function on CD8^+^ T Cell Subsets

Although Ly49 receptors are best known as innate immune receptors, their expression is not limited to the cells of the innate immune system which we have discussed above (Figure 1). Here, we will shift our attention to the Ly49-expressing T cells from the CD8 lineage, cells of the adaptive immune system. This population, along with the analogous KIR-expressing CD8^+^ T cells in humans, has only recently garnered much attention.

### CD8αα^+^ intestinal intraepithelial lymphocytes (IEL)

Ly49-expressing CD8^+^ T cells can be further classified based on whether they express a CD8αα or CD8αβ co-receptor. T cells expressing CD8αα and Ly49 receptors were identified among the IELs (10). Microarray analysis, confirmed by flow cytometry, comparing these cells to traditional intestinal CD8αβ T cells showed a marked increase in surface Ly49 expression, with CD8αα IELs possessing an Ly49 repertoire similar to – but distinct from – the NKT cells discussed above. Notably, although these IELs, like NKT cells, express predominantly inhibitory Ly49 receptors, the CD8αα T cells have also been shown to up-regulate Ly49D and H mRNA levels and display an increase in the activating DAP12 adaptor molecule, suggesting they may have some degree of activating Ly49 expression (10). Also, their Ly49 expression profile is remarkable based on its high levels of Ly49E and F, both of which are very rare in NK cell populations. Indeed, the bulk of CD8αα IELs are Ly49E and/or F positive, with the proportion of Ly49E/F double-positive cells roughly corresponding to the product of the proportions of single-positive cells (10). What role these cells may play in gut homeostasis is still unclear, although one report suggests that a subset of Ly49-expressing CD8αα IELs recognize the non-classical MHC-I molecule Qa-1 (HLA-E in humans) through their TCR and play an immune-regulatory role (117).

### CD8αβ^+^ T cells

In both humans and mice, the CD8αβ Ly49-expressing cells are mainly found among the CD122-expressing, memory-phenotype CD8^+^ T cells: CD25^−^Ly6C^+^CD44^+^ in mice, and CD28^−^CD45RA^−^CD45R0^+^ in humans (118). Despite being memory-phenotype and expanding with the age of the animal, these cells are present from an early age, suggesting that they are not true memory cells but a naturally occurring subpopulation of CD8^+^ T cells (119). As with NKT cells, they appear to express only inhibitory NK receptors. Additionally, like the CD8αα IELs, these CD8αβ T cells have non-NK-like Ly49 repertoires, in this case dominated by Ly49F expression in B6 mice (119).

Attempts to discern the purpose of inhibitory Ly49 or KIR expression on these cells have thus far identified two non-exclusive candidate functions. First, and most intuitive, expression and subsequent engagement of an inhibitory MHC-I receptor down-regulates killing activities from the T cell. Transgenic expression of the inhibitory Ly49A or KIR2DL3 on T cells inhibits their ability to kill MHC-mismatched target cells, provided that those targets express a ligand for the inhibitory NK receptor (120, 121). As this role for Ly49 or KIR expression is shared between T and NK cells, it is probable that it is for the same purpose: both CD8^+^ T cells and NK cells are directly lytic to self-cells while mounting an anti-viral or anti-tumor response, and so must have a method for healthy cells to be recognized and spared to avoid rampant autoimmunity (120). Especially in the case of regulatory T cells (discussed further below), Ly49 expression may allow for a population of CD8^+^ T cells with highly self-reactive TCRs that are regulated by inhibitory Ly49 receptors, analogous to NK immune surveillance. Such a model also suggests that T cells express only inhibitory Ly49 receptors because their TCR expression is performing a similar role as the activating receptor on the NK cell.

Interestingly, however, these inhibitory NK receptors appear to have a second function on T cells, conferring enhanced expansion and survival to the Ly49 or KIR positive cell. Although to date there is no study directly showing that Ly49 expression on mouse T cells confers enhanced IL-15 sensitivity, there are lines of evidence to suggest this. First, there is a marked correlation between Ly49 expression and IL-15 sensitivity on mouse CD8^+^ T cells (122). Using a simple CFSE proliferation assay, this report showed that, although many CD122-expressing T cells are IL-15 sensitive, those that also expressed Ly49 expanded most vigorously after IL-15 stimulation. Second, transgenic expression of a KIR on murine T cells causes those T cells, but not WT or KIR-expressing B cells, to possess a marked proliferation advantage when transferred into mice transgenic for the KIR ligand, but not when transferred into WT mice (118). Taken together, these reports strongly suggest that although Ly49/KIR expression may reduce a T cell’s cytotoxicity, it also enhances that cell’s longevity.

### CD8^+^ T cells with regulatory functions

Such a paradigm, where Ly49^+^ T cells are long-lived, broadly active, and/or highly self-reactive cells regulated by inhibitory Ly49 receptors, agrees conceptually with recent work from the Cantor lab and others demonstrating that Ly49-expressing CD8αβ ^+^ T cells include or entirely comprise a regulatory population implicated in preventing or controlling such autoimmune diseases as systemic lupus erythematosus (SLE), multiple sclerosis (MS), and rheumatoid arthritis (RA) (123–125). Much like the proposed regulatory CD8αα T cells above, these CD8αβ^+^ T_reg_ cells are characterized by a TCR restricted to the non-classical MHC molecule Qa-1 (HLA-E in humans) and reacting to a peptide derived from the HSP60 leader sequence (126). They act as indirect regulators of antibody production, by targeting the Qa-1-expressing follicular helper T cells (T_FH_) and lysing them in a perforin-dependent manner (127). Employing a strategy not unlike the NK cell use of inhibitory and activating Ly49, these CD8^+^ T_reg_ cells express NKG2A, an inhibitory NK receptor that recognizes Qa-1 presenting its dominant peptide, Qdm, which normally prevents their killing of T_FH_ cells. However, in cases of overactive T_FH_ giving rise to excessive antibody production by plasma cells, a shift toward more HSP60sp being presented by Qa-1, allows the CD8 T_reg_ cell’s TCR to compete with NKG2A and transmit an activating signal to the CD8^+^ T_reg_ cell. In a recent report, it was found that disrupting the ability for Qa-1 to interact with the CD8^+^ T_reg_ cell’s TCR – but not disrupting the NKG2A interaction – caused a massive expansion in T_FH_ and plasma cell numbers, splenic germinal center area, and serum and kidney antibody deposition, analogous to that seen in SLE-susceptible mice (127). Furthermore, adoptive transfer of these CD8^+^ T_reg_ cells into Rag^−/−^ mice with a reconstituted CD4^+^CD25^−^ T cell and IgM^+^ B cell compartment results in a suppression of antibody production upon immunization with NP, which appears to be mediated by the Ly49^+^, but not Ly49^−^ CD8^+^ T_reg_ cells (127). While no studies have yet shown a direct role for Ly49 in this regulation, the dual functions discussed above fit well with this model of regulation. On the one hand, the longevity and antigen-independent, IL-15-driven activation and proliferation of these T cells allows for their continuous presence and immunosurveillance within the organism in question; at the same time, the limiting effect of Ly49 and NKG2A, coupled with the scarcity of this CD8^+^ T_reg_ population, ensures that enough antibody is allowed to be produced to protect the host from infection.

## Conclusion

Members of the Ly49 family of receptors, like the human KIR, have a broader expression pattern than previously thought. Their expression is not restricted to NK and other cells of the innate immune system, but also found on cells of adaptive immune system. Similarly, their function seems to be broad and vary from regulation of target recognition by NK and possibly NKT cells, regulation of uNK cell functions during pregnancy, sensing of microbial macromolecules and stimulation of cytokine production by pDC, and modulation of autoimmune diseases by affecting survival and function of Ly49-expressing CD8^+^ T cells. The Ly49:MHC-I interactions are amenable to modulations with potential therapeutic agents, and the recent developments in understanding MHC-I receptor expression and function will aid in better design of such strategies.

## Conflict of Interest Statement

The authors declare that the research was conducted in the absence of any commercial or financial relationships that could be construed as a potential conflict of interest.
